# A joint transcriptional regulatory network and protein activity inference analysis identifies clinically associated master regulators for biliary atresia

**DOI:** 10.3389/fped.2022.1050326

**Published:** 2022-11-11

**Authors:** Panpan Sun, Manhuan Xiao, Huadong Chen, Zhihai Zhong, Hong Jiang, Xuyang Feng, Zhenhua Luo

**Affiliations:** ^1^ Department of Pediatric Surgery, The First Affiliated Hospital, Sun Yat-Sen University, Guangzhou, China; ^2^ Institute of Precision Medicine, The First Affiliated Hospital, Sun Yat-Sen University, Guangzhou, China

**Keywords:** pediatric, cholangiopathy, deconvolution, transcription factor, network

## Abstract

Biliary atresia (BA) is a devastating cholangiopathy in neonate. Transcription factors (TFs), a type of master regulators in biological processes and diseases, have been implicated in pathogenesis of BA. However, a global view of TFs and how they link to clinical presentations remain explored. Here, we perform a joint transcriptional regulatory network and protein activity inference analysis in order to investigate transcription factor activity in BA. By integration of three independent human BA liver transcriptome datasets, we identify 22 common master regulators, with 14 activated- and 8 repressed TFs. Gene targets of activated TFs are enriched in biological processes of SMAD, NF-kappaB and TGF-beta, while those of repressed TFs are related to lipid metabolism. Mining the clinical association of TFs, we identify inflammation-, fibrosis- and survival associated TFs. In particular, ZNF14 is predictive of poor survival and advanced live fibrosis. Supporting this observation, ZNF14 is positively correlated with T helper cells, cholangiocytes and hepatic stellate cells. In sum, our analysis reveals key clinically associated master regulators for BA.

## Introduction

Biliary atresia (BA) is an obstructive cholangiopathy with neonatal onset. Although incidence of BA is relatively low (1 in 5,000 to 1 in 14,000 live births) (1–3), it remains the leading indication for pediatric liver transplantation worldwide. The primary treatment of BA is a surgery intervention known as Kasai portoenterostomy (KPE). However, the efficient of KPE is limited and more than half of BA patients require liver transplantation after KPE. Thus, development of treatment for BA is essential for blocking disease progression and improving patient survival.

The etiology of BA is multifactorial. Prenatal insults such as genetic predisposition [eg. variants in ADD3 gene (4–6)], viral infection [e.g., cytomegalovirus (7, 8), rotavirus (9–11)] or toxins [e.g., biliatresone (12, 13)] engage the immune system and thereby induces an uncontrollable and amplifying innate and adaptive immune response, which manifests as infiltration of immune cells, activation of myofibroblasts, atresia of bile ducts and liver fibrosis (14, 15).

Among genes contributed to BA, multiple transcription factors (TFs) including FOXA2 (16), HNF1B (17), SOX17 (18) and ARF6 (19) have been identified, suggesting transcription factors served as key regulators in BA. For example, aberrant expression of SOX17 is associated with development defect of extrahepatic bile duct in mice and abnormal peribiliary glands in human BA patients (18). Transcription factors, a master regulator in diseases, modulate disease progression *via* several downstream transcriptional targets, forming a transcriptional network. Therefore, it is necessary to comprehensively uncover disease specific TFs and their target network to understand the global landscape of BA and to develop targeted therapeutic strategies (20, 21). Recent liver transcriptome analyses of human BA provide an opportunity to explore this in depth.

Several methods have been developed to reconstruct transcriptional network from transcriptome data (22–25). Among them, the algorithm for the reconstruction of accurate cellular networks (ARACNe) has been widely used in the research community due to its high accuracy (26). ARACNe reverse engineers transcriptional network by estimating mutual information between transcription factor and its targets. Despite high accuracy, ARACNe may not be able to identify disease specific TFs and transcriptional network as it does not consider differentially expressed genes between disease and normal control. Virtual inference of protein activity by enriched regulon analysis (VIPER) addresses this challenge by analyzing the expression of TF's entire targets (collectively referred to as TF's regulon) between disease and normal control, by taking into account the TF's mode of action, to infer TF's protein activity (27). The emergence of ARACNe and VIPER allow us to identify BA specific TFs and their downstream signaling.

In this study, by employing a joint ARACNe and VIPER analysis of three datasets of liver transcriptome (either RNA sequencing or microarray) of human BA, we identified 14 activated and 8 repressed transcription factors (or master regulators) for BA. Activated TFs target genes are enriched in SMAD, NF-kB and TGF-*β* pathways, while repressed TFs regulate genes are related to lipid metabolism. On the purpose of exploring the clinical association of TFs, we identified inflammation-, fibrosis- and survival associated TFs, with ZNF14 were predictive of advanced liver fibrosis and poor survival. Finally, we searched for the association between ZNF14 and cell infiltration, finding that ZNF14 was positively correlated with T helper cells, cholangiocytes and HSC.

## Materials and methods

### Samples description

We curated three liver transcriptome datasets (accession number: GSE122340, GSE46960 and GSE15235) from Gene Expression Omnibus (GEO) database (https://www.ncbi.nlm.nih.gov/geo/). Dataset I (GSE122340) contained 121 BA cases and 7 controls where the single-end RNA-seq data had been generated on an Illumina HiSeq 2500. Dataset II (GSE46960) contained 64 BA cases and 7 controls where the microarray data had been generated on GeneChip® Human Gene 1.0 ST Array. Dataset III (GSE15235) contained 47 BA cases where the microarray data had been generated on Affymetrix Human Genome U133 Plus 2.0 Array. The dataset III has clinical information of BA patients and is used for clinical analysis of transcription factors.

### Transcriptional network reconstruction

ARACNe (26), an information-theoretic algorithm for inferring transcriptional interactions, was used to identify candidate transcriptional regulators of the transcripts annotated to genes in dataset I, dataset II and dataset III. First, mutual interaction between a candidate TF (x) and its potential target (y) was computed by pairwise MI, MI (x, y), using a Gaussian kernel estimator. A threshold was applied on MI based on the null hypothesis of statistical independence (*P *< 0.05, Bonferroni-corrected for the number of tested pairs). Second, the constructed network was trimmed by removing indirect interactions using the data processing inequality (DPI), a property of the MI. Therefore, for each (x, y) pair, a path through another TF (z) was considered, and every path pertaining to the following constraint was removed: MI (x, y) < min [MI (x, z), MI (z, y)]. A *P* value threshold of 1 × 10−8 using DPI = 0.1 [as recommended (28)] was used when running ARACNe.

### Virtual protein activity analysis

The regulon enrichment on gene expression signatures was tested by the VIPER (27) (Virtual Inference of Protein-activity by Enriched Regulon analysis) algorithm in dataset I and dataset II. [Of note, dataset III cannot be used to estimate the protein activity because it does not provide normal controls (NC)]. First, the gene expression signature was obtained by comparing two groups of samples representing distinctive phenotypes, for example BA and NC samples in this study. In the next step, regulon enrichment on the gene expression signature can be computed using analytic rank-based enrichment analysis (29). At the end, significance values (*P* value and normalized enrichment score) were computed by comparing each regulon enrichment score to a null model generated by randomly and uniformly permuting the samples 1,000 times. The output of VIPER is a list of highly active MRs as well as their activity scores and their enrichment *P* values. Further information about VIPER can be accessed in (27). MRs with enrichment *P *< 0.05 by virtual protein activity analysis were identified as representative MRs of BA. Common MRs from dataset I and dataset II with enrichment *P *< 0.05 were selected MRs (Figure 2A).

### Functional enrichment analyses

Biological Process (BP) pathway enrichment analysis for selected MRs and for gene targets of selected MRs were conducted using ToppFun (https://toppgene.cchmc.org/enrichment.jsp; Transcriptome, ontology, phenotype, proteome, and pharmacome annotations-based gene list functional enrichment analysis, ToppFun). The maximum and minimum number of genes for each category were set to 2,000 and 5, respectively, based on the default setting. Bonferroni-Hochberg multiple test adjustment was applied to the enrichment output. FDR significance threshold was set to 0.05.

### Correlation between liver pathology, survival and expression of TFs

The matched liver grade and patient survival in dataset III were obtained from a previous study (30). In brief, inflammation grades were assessed by hematoxylin/eosin stain. According to the hematoxylin/eosin stain, no inflammation was considered grade 0, while portal expansion together with inflammation in >50% portal tracts was considered grade 3. Gomorra trichrome stain was used to assess the fibrosis grade, and no fibrosis was rated as stage 0, while portal fibrosis with expansion and bridging in >50% portal tracts or regenerative nodule was rated as stage 3. Inflammation/fibrosis grades were included in the correlation analysis. We performed Pearson correlation to evaluate the correlations between gene expression of TFs and inflammation/fibrosis grades in dataset III, and *P* value under 0.05 was considered significant.

The survival time was modeled by log-normal distribution. A Cox regression model was proposed to characterize the effects of selected MRs on liver native survival time. The cox regression was using the R package, survival (31).

### Correlation between immune cells, cholangiocytes, portal fibroblasts, hepatic stellate cells infiltration and protein activity of MRs

Abundance of immune cells, cholangiocytes, portal fibroblasts, hepatic stellate cells were estiamated by single sample gene set enrichment analysis (ssGSEA) using GSVA R package based on cell type specific signatures. The correlation between infiltrating immune cells scores and protein activity of selected MRs was estimated by Pearson correlation analysis.

## Results

### Transcriptional regulatory network and protein activity inference analysis of liver transcriptome identifies 14 activated and 8 repressed master regulators for BA

**Figure 1:**
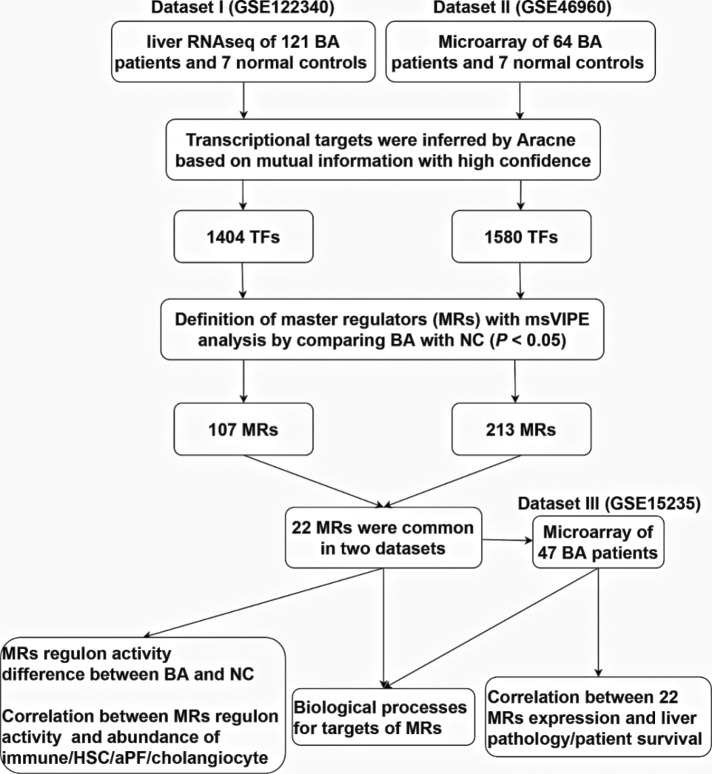
An outline of the systems biology analytical pipeline used in this study.

To comprehensively understand BA specific master regulators and their transcriptional regulatory network, we first developed an analytic pipeline started by analyzing of liver transcriptome of human BA (Figure 1). We included three liver transcriptome datasets of human BA from GEO database, which dataset I (GSE122340) contained liver RNA sequencing of 121 BA patients and 7 normal controls, dataset II (GSE46960) had liver microarray of 64 BA patients and 7 normal controls and dataset III (GSE15235) provided liver microarray of 47 BA patients and their clinical information (liver pathology and survival after KPE). To obtain high confidence master regulators and their downstream network, we used dataset I and II for deconvolution of the transcriptional regulatory networks and estimation of protein activity of master regulators, and used dataset III for clinical association analysis of master regulators (Figure 1).

**Figure 2:**
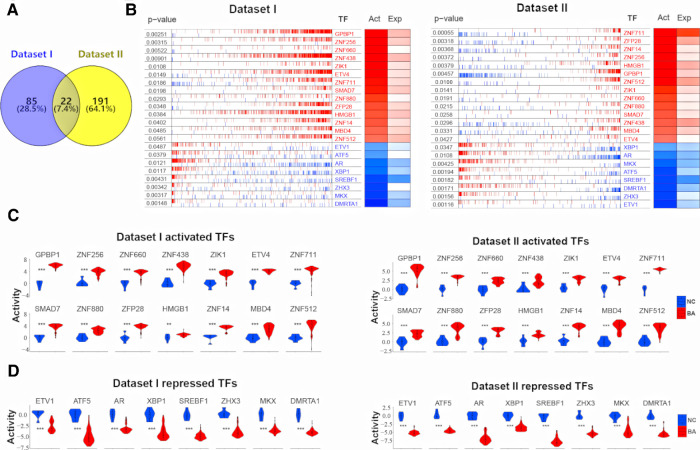
22 common TFs regulon activity in dataset I and dataset II. (**A**) Venn diagram shows 22 common TFs between dataset I and dataset II. (**B**) RNA expression profiles between biliary atresia (BA, dataset I, *n* = 121; dataset II, *n* = 64) and normal liver (NC, dataset I, *n* = 7; dataset II, *n* = 7) were evaluated for a transcriptional network activity through regulon analysis. Most differentially active 14 TFs-regulons were highlighted as red boxes and differentially repressed 8 TFs-regulons as blue boxes. (**C**) The violin plot demonstrated the difference of 14 activated TFs protein activity between BA (dataset I, *n* = 121; dataset II, *n* = 64) and NC (dataset I, *n* = 7; dataset II, *n* = 7). (**D**) The violin plot demonstrated the difference of 8 repressed TFs protein activity between BA (dataset I, *n* = 121; dataset II, *n* = 64) and NC (dataset I, *n* = 7; dataset II, *n* = 7).

Deconvolution of the transcriptional regulatory networks for dataset I and II with ARACNe identified 1,404 and 1,580 TFs, respectively (Figure 1). ARACNe first identifies gene-gene co-regulatory patterns by measuring mutual information, followed by network trimming through removing indirect relationships. We identified 1,404 TFs in dataset I, where the corresponding networks contain 16,646 targets and 249,801 transcriptional interactions (Supplementary Table S1). For dataset II, we observed 1,580 TFs and their regulatory targets in the constructed network, including 21,189 targets and 227,305 predicted interactions (Supplementary Table S2). Using the reconstructed transcriptional network, we next performed a virtual protein activity analysis of the TFs by considering the expression patterns of their downstream regulons between BA and normal controls through a dedicated probabilistic algorithm named VIPER. VIPER exploits the TFs mode of action, the confidence of the TFs-target gene interactions, and the pleiotropic features of the target regulation. We inputed the transcriptional network into VIPER to examine whether the TFs had a significant role in regulation of downstream target genes. VIPER outputs a short list of ranked TFs by adjusted activity *P* values and their potentially regulating a large set of targets (Supplementary Table S3). As a result, among the TFs with highly significant VIPER *P* values, we only took those with adjusted *P* < 0.05 as potential MRs. This process resulted in 107 potential MRs in dataset I (Supplementary Table S4) and 213 potential MRs in dataset II (Supplementary Table S5). We did not conduct virtual protein activity analysis in dataset III because of missing NC. Therefore, 22 TFs were common between dataset I and II (Figure 2A). All of these 22 TFs were significant in both datasets (*P* < 0.05). Of these 22 TFs, 14 TFs were upregulated in BA, whereas 8 TFs were downregulated when compared to NC (Figures 2B,C). We defined upregulated TFs as activated TFs while downregulated TFs as repressed TFs. Next, we processed to investigate the downstream signaling and clinical relevant for these TFs.

### Biological processes analysis reveals that enrichment of SMAD, NF-kappaB and TGF-beta signaling for targets of activated MRs and of metabolism for targets of repressed MRs

**Figure 3:**
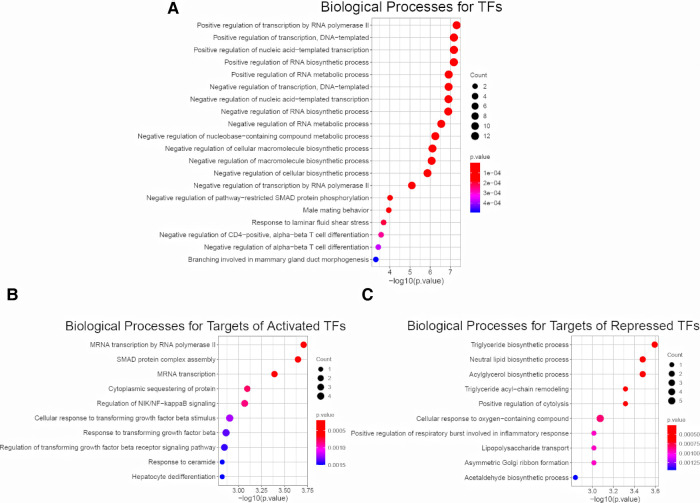
Enrichment analysis for 22 common TFs and their targets. (**A**) Biological Process Pathways for 22 common TFs. Pathway terms were ranked according to their −log10 (*p* value) values. The dot sizes were proportional to number of genes. (**B**) Top 10 Biological Process Pathways for targets of 14 activated TFs (dataset I, dataset II, dataset III). All the targets and pathways were common in three datasets. Pathway terms were ranked according to their −log10 (*p* value) values. The dot sizes were proportional to number of genes. (**C**) Top 10 Biological Process Pathways for targets of 8 repressed TFs (dataset I, dataset II, dataset III). All the targets and pathways were common in three datasets. Pathway terms were ranked according to their −log10 (*p* value) values. The dot sizes were proportional to number of genes.

To access the downstream mechanisms of these 22 TFs, we began by conducting biological processe analysis using ToppGene. A full list of enriched biological processes for 22 TFs was presented in Supplementary Table S6. As expected, top 10 enriched biological processes for these 22 TFs were related to transcription and RNA metabolism (Figure 3A). In addition, we observed other biological processes such as macromolecule biosynthesis (negative regulation of cellular biosynthetic process), immune associated pathways (Negative regulation of CD4−positive, alpha−beta T cell differentiation, Negative regulation of alpha−beta T cell differentiation), development/fibrosis (negative regulation of pathway−restricted SMAD protein phosphorylation, Branching involved in mammary gland duct morphogenesis) (Figure 3A). This initial result suggest that BA enriched TFs might be related to metabolism, immune response, development and fibrosis.

To further probe the downstream signaling, we fed the entire list of targets of 14 activated TFs for biological process analysis. We revealed several immune and fibrotic processes including SMAD protein complex assembly, Regulation of NIK/NF−kappaB signaling, response to transforming growth factor beta, were enriched (Figure 3B). This is consistent with activation of immune response and liver fibrosis in BA, and confirm that the activated TFs are master regulator. In contrast, the enriched processes for targets of 8 repressed TFs were largely linked to metabolism (Figure 3C). The findings demonstrate that immune and fibrosis regulating TFs are activated while metabolism regulating TFs are repressed in BA. This also in line with several previous observations that BA displayed abundant immune infiltration, advanced liver fibrosis and reduced liver metabolism compared to normal (14, 32–35).

### ZNF14 is associated with advanced fibrosis and poor survival

**Figure 4:**
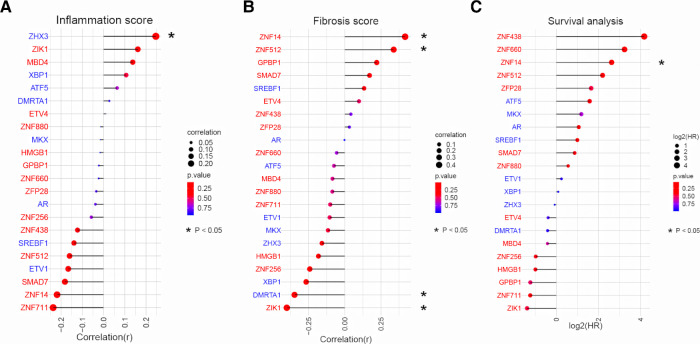
Correlation analysis between TFs expression and liver pathology/patient survival in BA. (**A**) Relationship between TFs expression and liver inflammation grade in BA liver. TFs were ranked according to the strength of the correlation (−1 < r < 1). The point size and line length represented the strength of the correlation. The color represented the *p* value. (**B**) Relationship between TFs expression and liver fibrosis grade in BA liver. TFs were ranked according to the strength of the correlation (−1 < r < 1). The point size and line length represented the strength of the correlation. The color represented the *p* value. (**C**) Cox regression for patient survival. TFs were ranked according to log2(HR). The point size and line length related to log2 (HR). The color represented the *p* value.

Having established the link between TFs and biological processes, we next sought to examine how they associate with clinical presentation. We used dataset III as it provided comprehensive clinical data (liver inflammation and fibrosis scores and survival time) for 47 BA patients. Pearson correlation analysis between expression of 22 TFs and clinical data showed that, only ZHX3 was positively associated with inflammation (Figure 4A). Activated TFs ZNF14 and ZNF512 were positively correlated with advanced liver fibrosis (Figure 4B). Survival analysis (cox regression) result revealed that only ZNF14 was predictive of poor survival. Taken all together, we conclude that ZNF14, an activated TFs associated with worse clinical outcomes, is a key MR in BA.

### ZNF14 predicts liver infiltration of T helper cells, cholangiocytes and hepatic stellate cells

**Figure 5:**
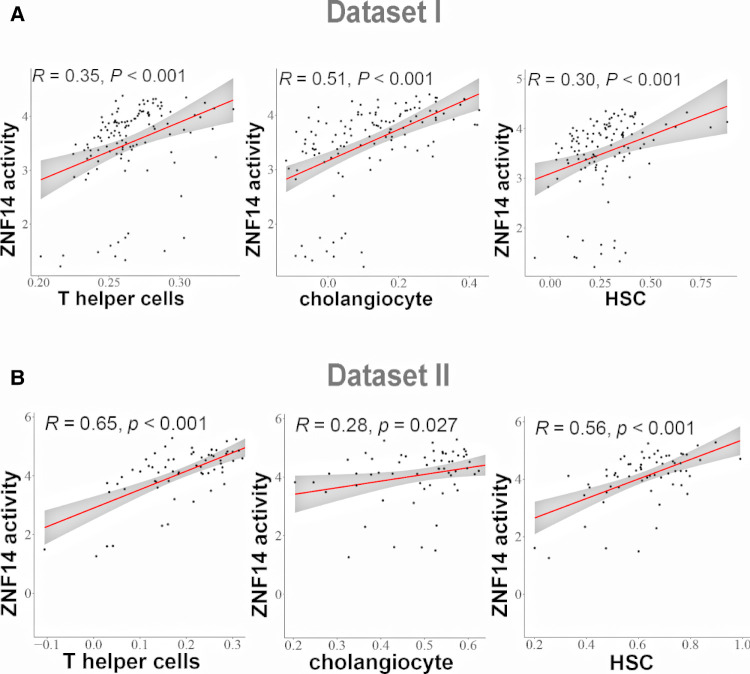
Correlation analysis between TFs regulon activity and single sample gene Set enrichment analysis (ssGSEA) scores. (**A**) Linear regression analysis between ZNF14 regulon activity and T helper cells/choanocyte/HSC ssGSEA scores (dataset I). (**B**) Linear regression analysis between ZNF14 regulon activity and T helper cells/choanocyte/HSC ssGSEA scores (dataset II).

Recent studies have shown that advanced liver fibrosis and poor survival is associated with infiltration of immune cells, cholangiocyte and hepatic stellate cells (36). ssGSEA was applied to access the immune cells/HSCs/PF/cholangiocytes infiltration in dataset I and dataset II. This analysis reveal several TFs correlated with cell infiltration. T helper cells and ZIK1, ZNF880, ZNF14, ZNF512, ETV1, XBP1 protein activity had the positive relationship, respectively (*P* < 0.05, Supplementary Figure S1A,C). Cholangiocytes and ZNF660, ETV4, ZNF711, SMAD7, ZFP28, ZNF14, ZNF512, ETV1, ZHX3 protein activity was positively related, respectively, while MBD4 was negatively related (*P* < 0.05, Supplementary Figure S1B,D). HSC was positively correlated with GPBP1, ZNF526, ETV4, ZNF880, ZNF14, ETV1, XBP1 protein activity respectively, while negatively correlated with ATF5 (*P* < 0.05, Supplementary Figure S1B,D). Among the examined cell types, the results of correlation analysis suggested that ZNF14 protein activity had positive correlation of T helper cells, cholangiocytes and HSC cells in dataset I and II (Figures 5A,B).

## Discussion

Like other complex disorders, BA is caused by multifactors (14). A major challenge for understanding the biological implications of master regulators is to confirm the core gene networks in BA-associated hepatobiliary tissue. In this study, with the aim of bringing to light the master regulators that may make contribution to BA pathogenesis, we have used an approach to assembling BA liver-specific transcriptional regulatory networks on two datasets (I and II). There were 22 MRs common in two datasets with *P* < 0.05. The confidence of the identified MRs was further strengthened by their presence in transcriptional profile and clinically validated by liver pathology and patient survival in dataset III.

Biological processes for 22 TFs were largely similar with those for gene targets of 22 TFs. Top 20 BP pathways for TFs were enriched in development, gene transcription, immune associated pathways, lipid metabolism. Biological processes for gene targets of activated TFs were relevant to development, gene transcription, immune associated pathways. In particular, for immune and fibrosis associated processes, they included regulation of NF-KB signaling, SMAD protein complex assembly, cellular response to transforming growth factor beta stimulus, response to transforming growth factor beta. These immune associated processes are probably key factors for BA. Many studies have demonstrated that these activities were involved in BA development. For example, SMAD activation was considered a characteristic feature of the TGF-*β* signaling pathway (37–40). NF-*κ*B was a key molecule in the expression of various inflammatory genes in antiviral innate immune reactions and thought to be implicated in the pathogenesis of BA (41–43). While gene targets for repressed TFs were enriched in BP pathways (top 10), most of them were about lipid metabolism, including neutral lipid biosynthetic process, triglyceride biosynthetic process, acylglycerol biosynthetic process. The repressed TFs and their targets were downregulated in BA, suggesting repressed TFs could promote lipid metabolism. As children with BA were at high risk for malnutrition, this data indicated that reduction of repressed TFs might contribute to worse progression of BA.

Searching for clinical association of 22 TFs, we found that ZNF14 was associated with advanced fibrosis and poor survival. Consistently, its protein activity was positively correlated to T helper cells, HSC and cholangiocytes cells infiltration. Supporting this observation, ZNF14 are mainly expressed in T cells, hepatic stellate cells, endothelial cells and erythroid cells in the liver single cell RNAseq data in human protein atlas database. Among related pathways for ZNF14 were gene expression (Transcription) and validated nuclear estrogen receptor beta network. Gene targets of ZNF14 were mostly enriched in BP pathways that were related to gene transcription/translation (mRNA Splicing-Major Pathway), cell cycle (regulation of cell cycle, cell cycle process), immune associated pathways (Innate Immune System, macro autophagy, regulation of complement cascade, regulation of response to stress), substate metabolism (peroxisomal lipid metabolism, metabolism of vitamins and cofactors, arachidonic acid metabolism) (Supplementary Table S7). Cell cycle pathways may be correlated with cholangiocyte abundance, while immune regulation activities may be involved in recruiting T helper cells and promoting the production of HSC. Thus, multiple lines of evidence from our study suggest that the ZNF14, possibly regulating biliary proliferation and bile duct development. Despite the possible effects of ZNF14 regulons on BA as discussed above, experimental validation is required to elucidate the function of ZNF14 in BA in the future.

In addition to ZNF14, other TFs suggest that there are additional gene networks contributing to BA. Among these identified TFs, SMAD7 and HMGB1 are two interesting factors. SMAD7 binds the E3 ubiquitin ligase SMURF2 in the nuclear and the binding complex translocates to the cytoplasm, in which TGF-*β* receptor type-1 interacts with SMAD7 and then they both degrade. Among its related BP pathways are regulation of transcription by RNA polymerase II, transforming growth factor beta receptor signaling pathway, negative regulation of epithelial to mesenchymal transition, BMP signaling pathway, positive regulation of cell-cell adhesion (Supplementary Table S8). Gene targets for SMAD7 are enriched in BP pathways about cellular response to growth factor stimulus, regulation of supramolecular fiber organization, substrate adhesion-dependent cell spreading, actin filament-based process (Supplementary Table S9). These pathways are closely associated with BA. HMGB1 has been reported to be associated with pathogenesis of BA (44–46). BP pathways for activated HMGB1 gene are related to negative regulation of transcription by RNA polymerase II, inflammatory response to antigenic stimulus and immune system process (Supplementary Table S10). In our deconvoluted gene network of HMGB1, its predicted targets were in the vast majority enriched in pathways related to cellular response to epidermal growth factor stimulus, positive regulation of transforming growth factor beta1, immune system development (Supplementary Table S11). The signal pathways descripted above have been proven to be altered in BA (44–46).

Some TFs, such as FOXA2, SOX17 have been reported to be the risk factors for BA. However, these TFs didn't reach significance in this study (Supplementary Table S12). One possibility is the dynamic change of expression of these TFs. These TFs may act at the very early phase of BA, and their expression change dramatically as the disease progress. The liver samples used in gene expression studies are unlikely at the very early phase. Thus, the previously reported TFs may not be captured in this study.

## Conclusion

In conclusion, using three datasets based on bioinformatics and clinical data, we identify a vital 22 master regulators related to BA. Of them, ZNF14 is likely a key factor which contributes to BA etiopathology.

## Data Availability

The original contributions presented in the study are included in the article/**Supplementary Material**, further inquiries can be directed to the corresponding author/s.
